# Genetic diversity and within-farm transmission of *Staphylococcus aureus* from ewes in Norway

**DOI:** 10.1186/s12866-026-04959-z

**Published:** 2026-03-20

**Authors:** Stefan Börjesson, Camilla Sekse, Siv Svendsen, Vibeke Tømmerberg, Annette Hegermann Kampen, Solveig Sølverød Mo

**Affiliations:** 1 Norwegian Veterinary Institute, Ås, Norway; 2 Animalia, Oslo, Norway

**Keywords:** Antimicrobial resistance genes, blaZ, Capsular polysaccharide, Commensal, Mastitis, Ovine, Sheep, Vaccination, Virulence genes

## Abstract

**Background:**

*Staphylococcus aureus* is the most commonly isolated bacterial species from ovine mastitis. Previous studies have mainly focused on dairy sheep, whereas knowledge about *S. aureus* in sheep kept for mutton and wool production remains limited. Furthermore, data on the occurrence of antimicrobial resistance genes and molecular epidemiology of *S. aureus* are scarce. In Norway, most sheep are kept for meat or wool, and mastitis caused by *S. aureus* results in substantial economic losses. This study aimed to determine the persistence, genetic relatedness and diversity, and antimicrobial resistance (AMR) of *S. aureus* in ewes at four farms in Norway using whole genome sequencing. The potential effect of vaccination on the occurrence of *S. aureus* with capsular polysaccharide type 8 (*cap8*) was also determined.

**Results:**

A total of 101 isolates from 70 ewes across four farms were characterized. Persistent colonization was observed in 24% of ewes. Among the isolates, 65% carried the capsular polysaccharide type 8 (*cap8*) gene and belonged to sequence types (STs) 8, 9, 49, and 8875. The remaining isolates were *cap5*-positive and belonged to STs 30, 130, 133, and 1640. No statistically significant differences were seen between isolates from vaccinated and unvaccinated ewes regarding the *cap* gene. Single-nucleotide polymorphism (SNP) analysis showed no evidence of inter-farm transmission but supported within-farm transmission. AMR genes were rare; the only clinically relevant gene identified was *bla*Z, present solely in ST8 isolates. Virulence gene profiling showed that the toxin genes *tst*-1, *sel*, and *sec* were common in ST133 and ST49 isolates and that no specific human-associated lineages could be detected.

**Conclusions:**

Despite the limited number of isolates and farms studied, the findings revealed high genetic diversity between farms, while one or two genotypes tended to dominate within a farm. AMR genes were uncommon, consistent with Norway’s low antibiotic usage and low prevalence of AMR. Most genotypes detected in this study have previously been described in ovine populations, indicating host-adapted strains. Vaccination appeared to have limited effect on the occurrence or distribution of *S. aureus* with *cap8*.

**Supplementary Information:**

The online version contains supplementary material available at 10.1186/s12866-026-04959-z.

## Background

During recent years the Norwegian sheep population has been 2.2–2.4 million individuals, with about 900.000–930.000 of those being breeding ewes (age > 1 year) [1]. Almost all sheep are kept for mutton meat and wool production with only a handful dairy herds. The population is divided among approximately 13.000 herds, with most of the herds being minor ones with < 100 individuals [2]. Compared to other European countries, the Norwegian production differentiates by that most herds are relatively small and kept on open field grazing during the summer but are then kept indoors during the rest of the year. In 2024, there were approximately 900.000 winter-fed sheep in Norway, and more than 1 million sheep carcasses approved for human consumption [2, 3]. The most common disease reported among ewes in Norway, and the main cause of economic loss for the farmers, primarily due to lower weight gain in lambs and culling of ewes, is mastitis [4]. It has been estimated that 6–7% of the ewes are affected by mastitis with occurrences ranging from 0 up to 30% within herds. Furthermore, it has been reported that approximately 20% of all ewes in the Norwegian Sheep Recording System (Sauekontrollen) were culled due to mastitis. From ewes with clinical mastitis in Norway, *Staphylococcus aureus* is by far the most common bacterial species isolated, identified in up to 65% of cases [4, 5]. Besides being an opportunistic pathogen, *S. aureus* is also commonly identified in the normal bacterial microbiota of ruminants, including ovine, and there appears to exist specific ovine-adapted genetic lineages [6, 7]. A Norwegian study conducted in 2006 in three mutton meat farms showed that the nasal carriage of *S. aureus* for ewes ranged from 41 to 67% in the herds, with only 1.5% positive in milk samples and 25% positive in teat swabs [8].

According to the Norwegian therapy recommendations, only acute mastitis in ewes should be treated with antibiotics with the primary option being benzyl-penicillin [9]. In total, 80% of all antibiotics prescribed to sheep are beta-lactamase sensitive penicillins, with > 90% of *S. aureus* isolates from sheep susceptible to benzyl-penicillin [10]. Overall antibiotic susceptibility reports specifically on sheep is scarce, but compared to other countries, it is likely that Norway has a very high fraction of susceptible strains [10]. Furthermore, the few published studies available from other countries are also almost exclusively on isolates from dairy farms but reports large variation in resistance to for example the penicillin-group of antibiotics, with up to 80% of isolates being resistant [11–15]. For example, a recent systematic review focusing on *S. aureus* from healthy livestock reported that the pooled prevalence of penicillin susceptibility in isolates from all studies on sheep and goats was only 65% [7]. In addition, MRSA has been reported from sheep from all over the world, including Norway where a *mec*C-MRSA ST130-t843 was detected in healthy sheep in 2018 [10, 11, 13, 14, 16]. MRSA has also been detected in Denmark which is another low prevalence country when it comes to antibiotic usage and resistance [17]. An alternative to antimicrobial treatment of mastitis is hindrance of the infection through vaccination. Although some studies have described reduction in intramammary infections and chronic infections, especially in combination with mastitis control programs, the vaccines do not prevent infection nor achieve complete clearance [18]. In addition, to our knowledge there seems to be a lack of extensive clinical field-studies. It is also worth mentioning that in human-medicine there are no successful vaccines against *S. aureus* available despite decades of research and efforts [19].

Despite sheep production being an important industry in Norway, there is a knowledge gap on the molecular epidemiology of *S. aureus* in ewes, the published studies available are few and relatively old, utilizing pulsed-field gel electrophoresis (PFGE) and focusing mainly on occurrence [5, 8, 20, 21]. The aim of the study was to determine persistence, genetic relatedness and diversity, and antimicrobial resistance (AMR) of *S. aureus* in ewes at four farms in Norway through whole genome sequencing. The potential effect of vaccination on the occurrence of *S. aureus* with capsular polysaccharide type 8 (*cap8*) was also determined.

## Methods

### Farms, samples and isolates

As part of a previous study focusing on if *S. aureus* vaccination might improve the udder health in ewes, milk samples were collected from a random selection of ewes at four different farms (A-D) in Norway during spring (May- June) 2022 (*n* = 607 ewes) and autumn (August-September) 2022 (*n* = 547 ewes). The 547 ewes sampled during autumn had also been sampled during spring and were thus longitudinally sampled. Samples were collected from both teats, if possible, but for 82 teats sampling was not possible. The farms were selected based on geographic location, size, membership in the Norwegian Sheep Recording System (Sauekontrollen) and the farmer’s willingness to participate in the study [22]. Three of the farms investigated were located in the same county of Norway, while the fourth was located in another county. Details regarding the four included herds are presented in Table 1. In the earlier study [22], a total of 103 samples were positive for *S. aureus*, and these samples were included in the current study. Detailed information on clinical scores was not available for inclusion in this study, but we know that some of the sampled ewes had clinical signs of mastitis [22]. Isolates included in this study may therefore originate from both healthy ewes and ewes affected by mastitis. Notably, all three ewes from Farm A sampled during summer pasture had clinical mastitis. At the request of the farmers and/or veterinarians at the farms, the farm and county have been anonymized for this study. The milk samples were stored at -20 °C. Milk samples positive for *S. aureus* were thawed, and ten µL milk per sample was spread on blood agar (Oxoid, blood agar base supplemented with bovine blood) and incubated at 37 °C overnight. Suspected *S. aureus* colonies were pure-cultured on blood agar and confirmed as *S. aureus* using MALDI-TOF (Bruker Daltonics).

**Table 1:** Number of samples and occurrence of *S. aureus* per farm. Overview of number of ewes sampled, number of *Staphylococcus aureus* isolates retrieved, number of ewes positive, number of ewes persistently colonized and number of ewes colonized in both teats, per farm

Farm	County	Size of farm	*n* of ewes sampled before grazing*	*n* isolates	*n* (%) number positive ewes before grazing	*n* of ewes sampled after grazing*	*n* isolates	*n* (%) number positive ewes after grazing	Total number of isolates	Total *n* ewes *S. aureus* positive	*n* persistent carriage	*n* ewes positive both teats spring and/or autumn
A*	X	301	161	14	13 (8.1%)	138	14	13 (9.4%)	31*	26	3	2
B	X	342	164	14	10 (6.1%)	153	21	17 (11.1%)	35	20	7	5
C	X	446	187	11	9 (4.8%)	173	14	13 (7.5%)	25	17	5	2
D	Y	274	95	4	3 (3.2%)	83	6	6 (7.2%)	10	7	2	1
Total			607	43	35 (5.8%)	547	55	49 (9.0%)	101	70	17	10

*An addition to sampling during spring and autumn, Farm A also provided samples from ewes with clinical mastitis during grazing (three samples from three different ewes). Overall, 31 isolates from Farm A

Size of farm = number of ewes with fetal counts

Approximately half of the ewes included in this study had been vaccinated against *S. aureus* mastitis Additional file 1 (Supplementary Table), using the VIMCO vet vaccine (Hipra) stimulating immunity against *S. aureus* with the capsular polysaccharide 8 (CP8), containing inactivated *S. aureus* strain SP 140 with CP8.

### DNA extraction and genome sequencing

Genomic DNA was extracted from fresh colonies using the QIAmp^®^ DNA Mini kit (Qiagen) following the manufacturer’s description for Gram-positive bacteria. After adding the lysis buffer containing lysozyme (Merck), 20mM Tris, 2mM EDTA, 1,2% Triton X-100, pH 8.0 and lysostaphin (SigmaAldrich), samples were incubated at 450 rpm and 37 °C at for 2 h. Also, RNAse A (100 mg/mL, Qiagen) was added to the samples after addition of Proteinase K and before addition of buffer AL. The DNA quality was determined using MySpec (VWR) and DNA quantity using a Tecan Spark Fluorometer (Tecan) with a Qubit broad range kit (Thermo Fischer Scientific).

Samples were prepared using the Illumina DNA Prep library preparation kit (Illumina) and sequenced on the MiSeq Illumina platform (Illumina) obtaining 300 bp paired-end reads or on a NextSeq 550 Illumina platform obtaining 150 bp paired-end reads (Supplementary Table 1 in Additional file 1).

### Bioinformatic, phylogenetic and statistical analysis

Initial quality control of the data was performed using the quality control pipeline in the in-house data analysis system VIGAS/P [23]. This included assessment of read quality using fastqc v 0.73 (https://www.bioinformatics.babraham.ac.uk/projects/fastqc/) and contamination check using Kraken2 v 2.1.3 [24] with the database Minikraken v2. Quality-controlled reads (for QC requirements, see Supplementary Table 2 in Additional file 2) were further analyzed to determine multi-locus sequence types (MLST) (github.com/tseemann/mlst) using the pubMLST typing scheme for *S. aureus* [25]. In addition, *S. aureus spa*-types were determined using spaTyper v 0.3.3 [26].

*De novo* assembly was performed using Shovill v 1.0.4 (github.com/tseemann/shovill) with default settings, and assembly quality was assessed using Quast v 5.0.2 [27]. Detection of the *cap5* and *cap8* loci was done using in silico PCR with published primers [28], while genes encoding AMR- and virulence factors genes was done using AmrFinderPlus v 3.11.18 with the –-plus option and default settings [29].

Phylogenetic analysis was performed with the ALPPACA pipeline [30] using the core_genome track to investigate the genetic relatedness within each sequence type (ST) per farm and the same STs across farms. The analysis was performed for all STs where five or more isolates were available from one farm. Also, if an ST was detected on three or four farms and five or more isolates were available, single nucleotide polymorphism (SNP) analyses were performed. A SNP alignment was reconstructed using parSNP v. 1.6.1 [31] using the longest assembly from the respective ST groups as reference in each analysis. Gubbins v. 3.1.6 [32] was used to identify recombinant regions using the GTRGAMMA model and RaxML as initial tree builder. Maskrc-svg v. 0.5 (https://github.com/kwongj/maskrc-svg) was used to mask putative recombinant regions before filtering using snp-sites v. 2.5.1 [33]. A maximum-likelihood phylogeny was made with IQ-TREE v. 2.2.0.3 [34]. The most suitable evolutionary model was identified using ModelFinder [35], and branch support evaluated using UFBoot2 [36]. Pairwise SNP distances between genomes were calculated with snp-dists v. 0.8.2 (https://github.com/tseemann/snp-dists) using the recombinant-filtered alignment. Phylogenetic trees were visualized and annotated with the ggtree package v 3.14.0 [37] in R v 4.4.3 [38].

To evaluate whether there was an effect of vaccination on the occurrence of *S. aureus* with *cap8* capsular polysaccharide, a two-sided Z-test was performed using the prop.test function in R v. 4.4.3 [39].

## Results

### Cultivation

*S. aureus* were isolated from 101 of the 103, previous positive, milk samples (98.1%). The samples originated from 70 different ewes (Table 1). Of the 101 isolates, 52 originated from vaccinated ewes and 49 from unvaccinated ewes. In both cases multiple samples from the same ewe could be positive (i.e. from both teats or samples collected both before and after grazing), but only a single isolate per teat per sample was included. Thus, for some ewes several *S. aureus* isolates were characterized. On each farm, a subset of ewes (17/70, 24.3%) was persistently colonized with *S. aureus* in one or both teats during both spring and autumn.

A complete overview of isolates and their genetic properties is presented in Supplementary Table 1 (see Additional file 1).

### Capsular polysaccharide, Multi-locus sequence typing (MLST) and *spa*-typing

Of the 101 isolates, 66 (65.3%) were positive for the *cap8* locus, while 35 (34.7%) had *cap5* (Table 2). Of the *cap8* isolates, 37 were isolated from vaccinated and 29 from unvaccinated ewes, while 15 and 20 *cap5* isolates were from vaccinated and unvaccinated ewes, respectively. No statistically significant difference (*p* > 0.05) between the occurrence of *cap5* and *cap8* in vaccinated vs. unvaccinated ewes was detected.

**Table 2:** Genotypes of *Staphylococcus aureus* isolated from ewes in four different farms in Norway. Genotypes of *Staphylococcus aureus* isolates (*n* = 101) originating from ewes at four different sheep farms in Norway, based on *cap*-type, multilocus sequence type and *spa*-type

Farm				A			B		C		D		
County				X			X		X		Y		
Capsular Polysaccharide (*cap*)	Clonal Complex	MLST	*spa*-type	Before grazing	Grazing (clinical mastitis)	After grazing	Before grazing	After grazing	Before grazing	After grazing	Before grazing	After grazing	Total
5	CC8	ST8	t1171	-	-	-	-	-	-	-	1	2	5
			t1476	-	-	-	-	-	-	-	1	1	
	CC9	ST9	tNew1*	1	-	1	1	-	-	-			3
	CC49	ST49	t208	4	-	7	-	-	1	1	-	-	24
			t1207	-	-	-	-	-	2	2	-	-	
			t7750	1	-	-	-	-	-	-	-	-	
			t11807	-	-	-	-	1	-	-	-	-	
			tNew2*	-	-	-	2	3	-	-	-	-	
	CC136	ST8775	t3853	-	-	-	-	-	-	-	2	1	3
8	CC30	ST30	t964	-	-	-	7	9	-	-	-	-	16
	CC130	ST130	t1773		-		-	-	6	8	-	-	14
	CC131	ST133	t2678		-		1	5	2	3	-	-	35
			t5592	-	-	-	-	-	-	-	-	1	
			t7302	-	-	-	-	-	-	-	-	1	
			t15249	4	2	3	3	3	-	-	.	-	
			tNew3/4*	4	1	2	-	-	-	-	-	-	
		ST1640	t4453	-	-	1	-	-	-	-			1
			Total	14		14	14	21	11	14	4	6	101

* Based on the genome sequencing and assignment of repeats using spaTyper tNew1: 07-16-23-02-12-23-02-20-34 (*n* = 3), tNew2: 04-20-17-17-31-31-24-17-17 (*n* = 5), tNew3: 03-16-12-437-17-23-13-17-17 (*n* = 6), tNew4: 03-12-437-17-23-13-17-17 (*n* = 1)

From each farm, A-D, multiple STs could be detected with three to four STs per farm identified (Table 2). For ST9, ST30, ST130, ST1640 and ST8775, only one *spa*-type per ST was identified, while ST8, ST49, ST133 belonged to multiple *spa*-types. Four new *spa*-types were identified in the study which hereafter referred to as tNew1 to tNew4, where tNew1 belonged to ST9, tNew2 to ST49, tNew3 and tNew4 to ST133.

### Comparison across farms

In the four farms included multiple STs were identified, although at each farm one or two STs predominated. ST9, ST49 and ST133 were identified in two or more farms (Table 2). Only three isolates from two farms were identified as ST9 and they were all *spa*-type tNew1. ST49 were identified in combination with four different *spa*-types, but only ST49-t208 were identified in more than one farm. ST133 were identified in combination with five different *spa*-types, with ST133-t2678, and ST133-t15249 identified in two farms each (Table 2).

**Fig. 1:**
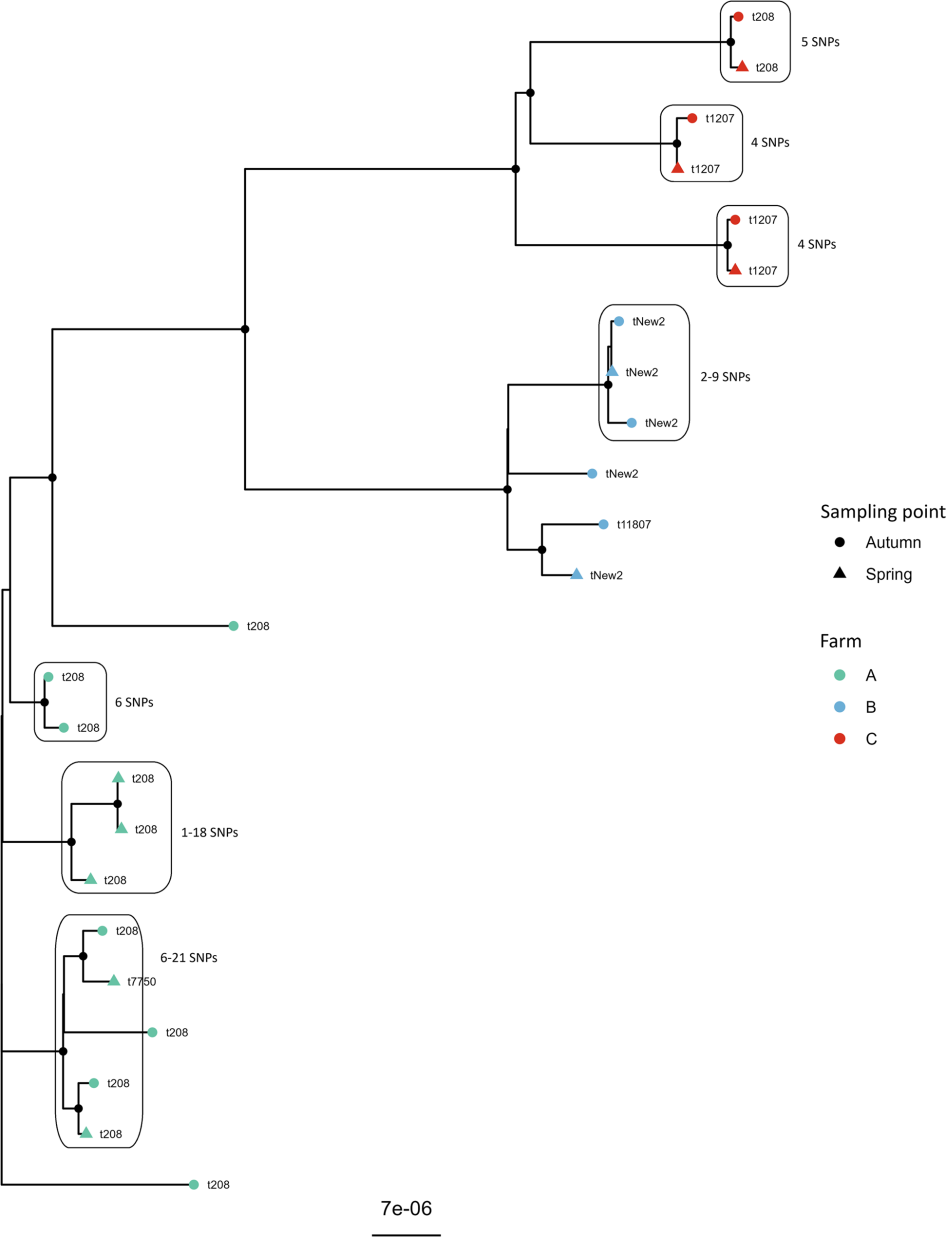
Core genome SNP tree of 24 *Staphylococcus aureus* multilocus sequence type 49. The isolates originate from ewes at three different farms in Norway. Tip-points are colored according to farm of origin, while the shape of the tip depicts the sampling point. SNP ranges are indicated for closely related isolates

SNP analyses of ST49 across farms (Fig. 1) revealed a minimum of 156 SNPs between isolates from Farms A and B. For isolates from Farms A and C, a minimum of 182 SNPs was observed, although some isolates had the same *spa*-type. Isolates from Farms B and C differed by a minimum of 198 SNPs with no overlapping *spa*-types between farms (Table 2).

**Fig. 2:**
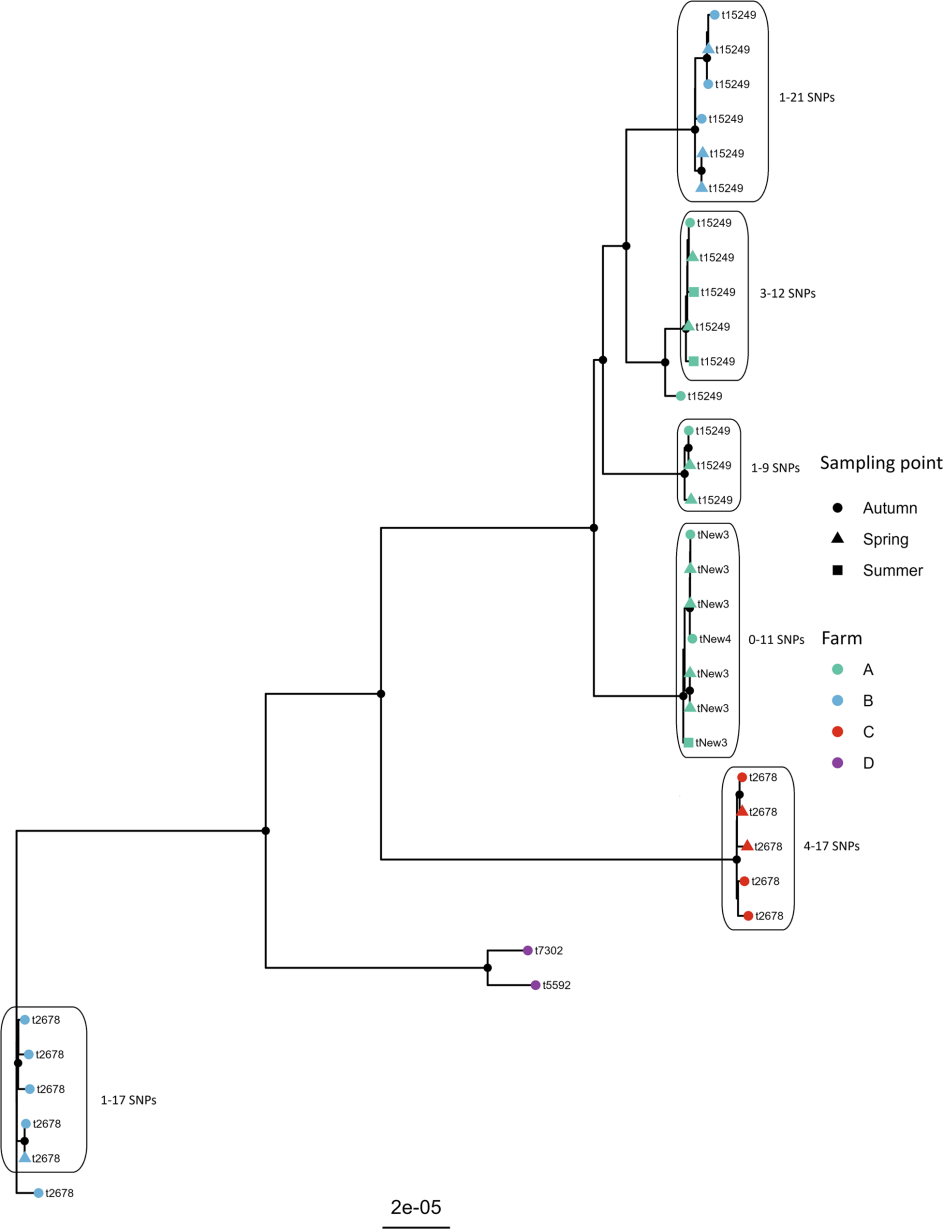
Core genome SNP tree of 35 *Staphylococcus aureus* multilocus sequence type 133. The isolates originate from ewes at four different farms in Norway. Tip-points are colored according to farm of origin, while the shape of the tip depicts the sampling point. Isolates collected during summer are from ewes with clinical mastitis. SNP ranges are indicated for closely related isolates

For ST133, overlapping *spa*-types were only observed between Farms A and B and Farms B and C, respectively (Table 2; Fig. 2). Isolates from Farms A and B differed by a minimum of 100 SNPs, while isolates from Farms B and C differed by a minimum of 518 SNPs. For the remaining Farm combinations, a minimum of 400 SNPs were observed between isolates (Fig. 2).

### Within farm comparison

#### Farm A

A total of 31 *S. aureus* isolates were retrieved from 26 ewes, belonging to ST9, ST49, ST133 and ST1640 (Table 2).

ST9-tNew1 was isolated from two different ewes, and ST1640-t4453 from a single ewe.

ST49 were isolated from ten different ewes. One carried ST49-t208 at both sampling occasions differing by 7 SNPs, while another was colonized with ST49-t7750 during spring and ST49-t208 during autumn, differing by 12 SNPs. The remaining eight ewes carried ST49-t208, including one ewe also positive for ST133-t15249 in the other teat. Overall, ST49 isolates from Farm A differed by 1-112 SNPs. Highly similar ST49-t208 isolates were isolated from different ewes (Supplementary Fig. 1, see Additional file 3).

ST133, belonging to *spa*-types t15249, tNew3 or tNew4 (Table 2), was found in 14 different ewes, including three ewes with clinical mastitis during summer. These three were not colonized during spring or autumn. The *S. aureus* from the three clinically affected ewes belonged to ST133-t15249 (*n* = 2) and ST133-tNew3 (*n* = 1) and differed by 16–166 SNPs. One ewe was positive both spring and autumn, and the isolates were ST133-t15249 differing by 7 SNPs. Another ewe was positive in both teats during spring. These isolates were ST133-tNew3 and no SNPs were detected. Overall, up to 171 SNP differences were observed among the isolates, but sub-clusters of highly similar isolates from different ewes were observed (Supplementary Fig. 2, see Additional file 3).

#### Farm B

A total of 35 *S. aureus* isolates were retrieved from 20 ewes, which belonged to four different STs, namely ST9, ST30, ST49 and ST133 (Table 2).

ST30-t964 was present in eight ewes (Supplementary Fig. 3, see Additional file 3). Five of these were positive both spring and autumn, and/or were colonized in both teats. Up to 122 SNP differences were observed for the ST30-t964 isolates. Isolates from the same individual were closely related, differing by 0–23 SNPs. However, three isolates from one ewe colonized spring and autumn differed by up to 34 SNPs.

ST49 was isolated from four different ewes, two of which were positive both spring and autumn. The isolates belonged to two different *spa*-types, t11807 and tNew2 (Table 2). Isolates from one of the ewes colonized both spring and autumn had *spa*-types t11807 and tNew2 and differed by 24 SNPs, while isolates from the other ewe differed by eight SNPs. Overall, ST49 isolates differed by 3–55 SNPs (Supplementary Fig. 4, see Additional file 3).

ST133 was present in ten different ewes and belonged to *spa*-types t2678 and t15149 (Table 2). Two ewes were positive both spring and autumn with one carrying ST133-t2678 with no SNP differences, and the other carrying ST133-t15249 differing by 7 SNPs. Overall, the ST133 isolates showed > 500 SNP differences, but also grouped into two distinct sub-clusters based on *spa*-type. ST133-t15249 isolates differed by 1–22 SNPs and ST133-t2678 isolates differed by 0–30 SNPs (Supplementary Fig. 5, see Additional file 3).

Five ewes were colonized in both teats. Two had ST30-t964 in both teats, one had ST30-t964 in one teat and ST133-t2678 in the other, and one had ST49-tNew2 in one teat and ST133-t2678 in the other. The last ewe had ST30-t964 in one teat at both sampling occasions, and ST49-tNew2 and ST49-t11807 in the other teat during spring and autumn, respectively.

#### Farm C

Three different STs, namely ST49, ST130 and ST133 were present among 25 *S. aureus* isolates from 17 ewes on Farm C (Table 2).

ST49-t1207 and ST49-t208 were detected in two and one ewe, respectively, with all three being positive at both sampling occasions. Isolates from the same ewe differed by < 10 SNPs, while > 90 SNPs was observed between individuals, irrespective of *spa*-type (Supplementary Fig. 6, see Additional file 2). One ewe carried ST49-t208 in one teat and ST130-t1773 in the other. In total, ST130-t1773 was isolated from 11 different ewes. One ewe was colonized in both teats during spring and autumn, and the four isolates collected from this ewe differed by 1–5 SNPs. In seven other ewes, closely related isolates, differing by 0–13 SNPs were detected. Overall, the ST130-t1773 isolates differed by 1–46 SNPs (Supplementary Fig. 7, see Additional file 3).

ST133-t2678 was isolated from four ewes, of which one was colonized both spring and autumn, with isolates differing by 6 SNPs, while ST133-t2678 isolates differed by 6–21 SNPs overall (Supplementary Fig. 8, see Additional file 3).

Besides the ewe which carried ST130-t1773 in both teats, one additional ewe was positive in both teats with ST49-t208 in one teat and ST130-t1773 in the other.

#### Farm D

From Farm D, 10 *S. aureus* isolates were retrieved from seven different ewes, belonging to ST8, ST133 and ST8775 (Table 2). ST8-t1476 and ST8-t1171 were detected in two and one ewes, respectively, and two were positive both spring and autumn. One ewe carried ST8-t1171, with isolates differing by 13 SNPs, while the other carried ST8-t1476 differing by 32 SNPs. The ST8-t1171 isolate from the third ewe differed by ≥ 170 SNPs from the other isolates, and this ewe also carried ST8775-t3853 in the other teat. Overall, the ST8 isolates differed by 13–223 SNPs (Supplementary Fig. 9, see Additional file 3).

*S. aureus* ST133 were isolated from two ewes, one with *spa*-type t5592 and one with t7302. Further, ST8775-t3853 were isolated from three different ewes.

### Antimicrobial resistance genes

The *blaZ* gene encoding penicillinase was detected in ST8 isolates (*n* = 5, 5.0%, Farm D). In all isolates genetic markers proposed to be associated with fosfomycin were identified. These were the *fos*B gene (ST8, ST9, ST8775, ST30, ST133), the chromosomal mutations *glp*T_A100V, *mur*A_E291D, and *mur*A_T396N (ST49, ST8775, ST30, ST130, ST133, ST1640), and glpT_V213I, murA_D278E (ST30). In addition, in all isolates the intrinsic *tet(*38) gene was detected.

The arsenic resistance gene *ars*B was identified in all ST130 isolates.

A full overview of AMR genes detected is presented in Supplementary Table 1 (Additional file 1).

### Virulence genes

All isolates carried the *aur* and *hlg*C genes. The *ica*C gene, part of the *ica* cluster, was also detected in all isolates, but no isolates harbored the other genes, *ica*ADB, associated with the cluster. Further, *hlg*B was detected in the majority of isolates (97/101, 96,0%).

No isolates contained the human immune evasion cluster (IEC) (6) genes *scn*, *sak* and *sea*/*sep*, nor the Panton-Valentine leucocidin (PVL) toxin genes, *luk*S-PV *and luk*F-PV. All ST8 isolates were positive for *luk*D and *luk*E *genes*, encoding leukotoxins, while the *luk*E gene was also identified in all ST49, ST8775, ST130, ST133, ST1640 isolates (*n* = 77, 76%).

The genes *tst*-1 *(*encoding toxic shock syndrome toxin), *sel*, and *sec* (encoding staphylococcal enterotoxins) were associated with ST133 (28/35, 80%) and ST49 (12/24, 50%). These genes were only identified in isolates from Farms A and B and connected to specific *spa*-types (Table 2 and Supplementary Table 1).

All isolates were positive for at least one enterotoxin/-like gene with the three ST9 isolates positive for *sel*26, *sel*27, *sel*28, *sel*X, *sey*,* seo*,* sen*,* seu*,* sei* and *sem*. The genes *sel27*,* sel28*, *sey* and *seo* were not identified in any other STs, while *sen*,* seu*,* sei* and *sem* were also identified in ST30 (*n* = 16). The *sel*X was detected in all STs, with the exception of ST30 isolates, and ST30 also lacked the *sel*26 gene, which were neither detected in ST130 nor ST133. The serine protease genes, *spl*A and *spl*B, were not identified in ST30, nor in the ST9, but were detected in all other STs. All ST30 isolates did however carry the *spl*E gene, as did all ST49, ST8775, ST30, ST130 and ST1640, while it was not detected in ST133, ST8 and ST9.

The majority of ST30 isolates (*n* = 15/16, 93.8%) was positive for the collagen adhesion gene *cna*, and all ST130 carried the epidermal cell differentiation inhibitor gene *edn*B, these two genes were no identified in the other STs.

A full overview of virulence and toxin genes detected is presented in Supplementary Table 1 (Additional file 1).

## Discussion

To our knowledge this is the first study comparing *S. aureus* isolates using WGS from longitudinally sampled ewes and evaluating the genetic similarity within and between sheep farms in Norway. Several different STs, with isolates representing both *cap5* and *cap8* genotypes, were detected at each farm, and generally one or two STs predominated within each farm. Although only ten isolates were included from Farm D, ST8 constituted half of the isolates there. When overlapping STs were observed across farms, isolates generally belonged to different *spa*-types at different farms (Table 2). One should however be aware that *spa*-types based on assembly of short-read WGS-data can be misconstrued, especially if identical repeats are in long series [26]. For example, there is a possibility that ST49-t208 and ST49-t1207 could be the same *spa*-type, as t1207 differ only in one extra repeat compared to t208. Based on STs and *spa*-types, there was no evidence supporting that *S. aureus* transmission has occurred recently between the investigated farms in Norway, nor that a dominating specific clonal linage has established itself in the Norwegian sheep population. This is further supported by the SNP analysis where a high number of SNPs was seen for isolates of the same STs between farms. The results do however indicate that within farm transmission of *S. aureus* has occurred, which was also indicated in a previous Norwegian study utilizing PFGE [8]. Geography might also be a factor when investigating the *S. aureus* epidemiology, since the only farm located in another county (Farm D) differed significantly in its *S. aureus* population. At Farm D, ST8, which carried the *bla*Z, and ST8875 were the most common STs and they were only detected at this farm. This observation might also be due to other factors, i.e. antimicrobial usage at specific farms and farm management factors not investigated in this study or be influenced by the limited number of *S. aureus* isolates from this farm. In addition, ST30 and ST130, ST1640 were also only detected at one farm each, Farm B, C and A respectively. Overall, these results concur with earlier Norwegian studies utilizing PFGE showing between farm differences and several strains within in each farm, but generally also with one dominating type per farm [8, 20, 21].

When comparing isolates within farms, a large variation in SNPs could be observed, and some STs also differed in *spa*-types and virulence gene profiles. These findings may indicate that several different lineages were introduced on several occasions and circulate simultaneously within a farm. On the other hand, ST133 isolates from Farm C were conserved with only 6–21 SNPs and only one *spa*-type identified, compared to Farms A and B where several *spa*-types and up to 564 SNP differences were present. These findings emphasize the differences in the *S. aureus* epidemiology within different farms. Multiple introductions of ST133 may have taken place on Farms A and B, with different ST133 strains circulating for a prolonged period. Alternatively, an early establishment of a specific strain which then diverged through subsequent evolution at Farms A and B, respectively, might also explain the large difference. However, the number of SNPs and the distinct *spa*-types make this explanation less likely. On the other hand, Farm C seems to have a specific ST133 “house-strain” colonizing several ewes, or potentially only one recent introduction to the farm. Based on PFGE, a previous study indicated that a small number of related genotypes circulated nationally in ewes with mastitis in Norway, and that these clustered to a large degree with mastitis isolates from cows and goats [20]. Furthermore, a French study also utilizing PFGE concluded that identical or highly related strains circulated in five farms [39]. However, PFGE will underestimate the diversity of *S. aureus*, especially for distinguishing isolates within major lineages [40–42]. Despite STs (which can roughly be correlated to PFGE clusters) overlapping, our results show that the *S. aureus* population is more diverse between farms and hosts than previously indicated. This is further indicated by international studies [7], but additional national and international studies are needed to confirm this assumption.

Ewes positive for *S. aureus* in the same teat on both sampling occasions were generally colonized with the same ST-*spa* combination, or closely related *spa*-types, both spring and autumn. In addition, the isolates generally carried the same virulence factors and differed by a limited number of SNPs. In four out of ten ewes with identification of *S. aureus* in both teats, the isolates were also closely related, differing by a limited number of SNPs. These observations suggest persistent colonization with the same *S. aureus* strain in some ewes. However, in three ewes colonized in the same teat at different sampling occasions, the isolates differed by 24–34 SNPs. This could indicate that they were re-colonized with a slightly different strain via transmission within the farm. Other factors might also have influenced the SNP differences in these isolates. For example, a considerable variation in *S. aureus* diversity within the same host was described during an MRSA outbreak in a veterinary hospital [43]. In a previous study evaluating transmission and microevolution of MRSA in households also described a mean number of SNPs varying from 12-18 was reported, with strains likely persisting within the household for >2 years 44]. One should also be aware that *S. aureus* has been estimated to have mutation-rates ranging from 2.0 to 5.8 SNPs per year when colonizing the same host [45, 46]. Due to this host variation, microevolution and natural mutation rate it has been suggested that when investigating *S. aureus* outbreaks a cut-off of at least 20–28 SNPs should be considered [47–49]. Furthermore, it should be noted that only a single isolate was characterized per teat, therefore it cannot be ruled out that ewes are simultaneously colonized by several *S. aureus* strains in the same teat. For example, six ewes colonized in both teats were found to carry different STs, which could indicate co-colonization of multiple strains.

Despite the fact that the Norwegian sheep production distinguishes itself from other countries, the *S. aureus* population shows similarities to those reported from other countries and production types. This is likely due to existence of specific ovine adapted lineages, which have been established in the population for a long time [7]. These can also carry pathogenicity islands and prophages with host specific toxin genes such as *tst*,* sel*, and *sec*. For example, in our study, ST133 was strongly associated with the virulence genes *tst*,* sel*, and *sec.* This ST has also been frequently reported from ovine populations around the world, with the overlap extending to specific *spa*-types [16, 50–55]. However, the second most frequent ST in the current study, namely ST49, has to our knowledge not been described previously in small ruminants. Potentially the occurrence could be due to transmission from wildlife, as ST49 are frequently identified in different wildlife species, but it has also been associated with MRSA in pigs in Switzerland [56–60]. However, at Farm A all isolates were also positive for the genes *tst*, *sel* and *sec*, indicating ovine host adaption [6]. Of the other STs identified, all have been described previously in ovine, except for ST8775 and ST30 [16, 17, 50–55, 61, 62]. ST8, ST9 and ST30 have also been shown to have a broad host range, including other ruminants and humans. However, as none of the isolates in this study carried toxin genes associated with the human IEC [6], suggesting that human spill-over was limited. There was also no clear difference observed in the *S. aureus* genotypes from ewes sampled in spring form those reported with clinical mastitis during summer, which is in line with a recent Dutch study [50]. However, only three isolates from ewes with clinical mastitis were included in our study.

The detection of acquired genes encoding antibiotic resistance was limited, with only *bla*Z and *fos*B detected, and *blaZ* only present in ST8 isolates. However, the identification of *fos*B in 62 isolates, and mutations causing fosfomycin resistance in most isolates and STs was unexpected, as fosfomycin is not used in Norwegian livestock. As no phenotypic testing was performed, it is uncertain if they actually induced reduced fosfomycin susceptibility. In case of the *fos*B gene, it has also been shown to be frequent in *Staphylococcus spp.* from human and cattle in Sweden, where fosfomycin use is also non-existing, with all ST133 *S. aureus* isolates positive for this gene [63, 64]. In fact, *fos*B appears in literature to be almost ubiquitous connected to ST133, ST8 and ST9 [52, 54, 65, 66]. Thus, it can be postulated that the detection of *fos*B is associated with specific STs rather than selective pressure.

In this study about half of the ewes had been vaccinated against *S. aureus* with *cap*8 using VIMCO vet (Hipra). Administration of this vaccine has been described to give less severe mastitis [67–71]. However, our results indicated limited effect on the occurrence of genotypes as there was no statistically significant difference in occurrence of *S. aureus* with *cap5* or *cap8* in vaccinated and unvaccinated ewes. In fact, most identified isolates had the *cap8* locus regardless of the vaccine statuses. In addition, one vaccinated ewe developed clinical mastitis caused by *cap*8-ST133-tNew3, and this isolate was closely related to those from other ewes at the same farm. Furthermore, no significant differences on occurrence of *S. aureus* in milk samples, clinical signs of mastitis or growth of lambs was seen in vaccinated compared to unvaccinated ewes in a recent study [68]. Despite there being no association between vaccination and distribution of *cap*5/8, nor on the dominating STs, it’s interesting to note that all ST8 (*cap5*, Farm D), and 94% of ST30 (*cap*8, Farm B) isolates were from vaccinated ewes. These STs carried a wider set of genes encoding toxins, and the enterotoxins identified are generally associated with vSaβ staphylococcal pathogenicity islands (PAI) [72]. This might indicate that the vaccination potentially may have favored more pathogenic strains, although we only investigated a limited number of isolates. However, this observation still warrants further investigations to determine if this is an effect of the vaccine, or just a coincidental observation. Furthermore, we cannot rule out that vaccination influenced the severity of mastitis in vaccinated ewes or the immunological reaction to *S. aureus* with *cap8*, as this was not addressed in this study. It is also important to remember that only a limited number of ewes from a limited number of farms were included in the study.

The current study also raises several questions that should be addressed in future research. For example, longitudinal studies should be considered which would improve our understanding of host-adaption, transmission dynamics, introduction of new genotypes, and verify whether certain ewes are true persistent carriers, and if the same strains persist between breeding seasons. This information is needed to better understand the role of persistent carriers and specific strains in recurrent and endemic mastitis. The reasons for this are that some ewes in this study appears to be persistently colonized by the same strain, and certain strains seem to persist on the farms. In addition, some ewes appear to be colonized with different strains over time or at the same time. One specific limitation of the study is also the lack of comparison to clinical signs of acute or chronic mastitis and genotypes, which is needed to get a better understanding if specific genotypes are more prone to cause mastitis. This information is also required to elucidate any potential clinical effect of vaccinations against *S. aureus*. However, this study and others indicate that host-factors, within farms transmission and carriage are the most likely drivers of mastitis at a specific farm, but longitudinal studies could provide further support to this notion. Additional studies on host and environmental factors should also be considered to better understand their role in the development of clinical mastitis caused by *S. aureus*, especially related to persistent carriage.

## Conclusions

Comparison of *S. aureus* across farms revealed high genetic variability, and no indication of a common source for these bacteria. Thus, there must be multiple sources for *S. aureus* colonizing the udder of Norwegian ewes. Further studies are therefore warranted to shed light on the bacterial dynamics and epidemiology within and across sheep farms, and to determine effective preventive measures to limit the economic loss and animal welfare concerns caused by mastitis. However, some animals are also likely colonized by *S. aureus* over time and certain specific genotypes are transferred between animals within a herd. Hence persistent carriers could therefore be a potential target for mastitis prevention and control. However, interpretation of the results in our study should been done carefully as the number of isolates, ewes and herds are limited. Although despite the limited number of isolates and farms studied, the findings in this study revealed high genetic diversity between farms, while one or two genotypes tended to dominate within each farm.

## Supplementary Information

Additional file 1: Excel sheet, xlsx. Supplementary Table 1. Including information for all 101 isolates included in the study. Information include animal ID, farm ID, sampling point, teat, multilocus sequence type, spa-type, cap-type, vaccine status, presence/absence of genes encoding virulence traits or antimicrobial resistance, as well as the sequencing machine used for whole genome sequencing of the selected isolate.

Additional file 2: Word file, docx. Supplementary Table 2. Including an overview of minimum QC requirements for sequencing data.

Additional file 3: Word file, docx. Supplementary Figures. Illustrating core-genome SNP trees of Staphylococcus aureus of a given multilocus sequence type from ewes at a single farm.

## Data Availability

Raw reads are available from the European Nucleotide Archive (ENA, accession numbers PRJEB102579). The dataset supporting the conclusions of this article is presented in the supplementary table. Isolates and any additional data potentially not included in the article or in the supplementary material are available from the authors upon reasonable request.

## Abbreviations

*cap*5/8: Capsular polysaccharide types 5 and 8 genotype
MRSA: Methicillin-resistant *Staphylococcus aureus*
MLST: Multi-Locus Sequence Typing
PFGE: Pulsed-Field Gel Electrophores
SaPI: Staphylococcus aureus pathogenicity island
ST: Sequence type
SNP: Single nucleotide polymorphism
WGS: Whole genome sequencing
