# Supplementary material for: Genetic diversity and within-farm transmission of Staphylococcus aureus from ewes in Norway
Source: BMC Microbiol. 2026 Mar 20;26:412. doi: 10.1186/s12866-026-04959-z (PMC13126708; doi:10.1186/s12866-026-04959-z)
Supplement: Supplementary file 3 — Additional file 3: Word file, docx. Supplementary Figures. Illustrating core-genome SNP trees of Staphylococcus aureus of a given multilocus sequence type from ewes at a single farm. [file 12866_2026_4959_MOESM3_ESM.docx]

Supplementary figures


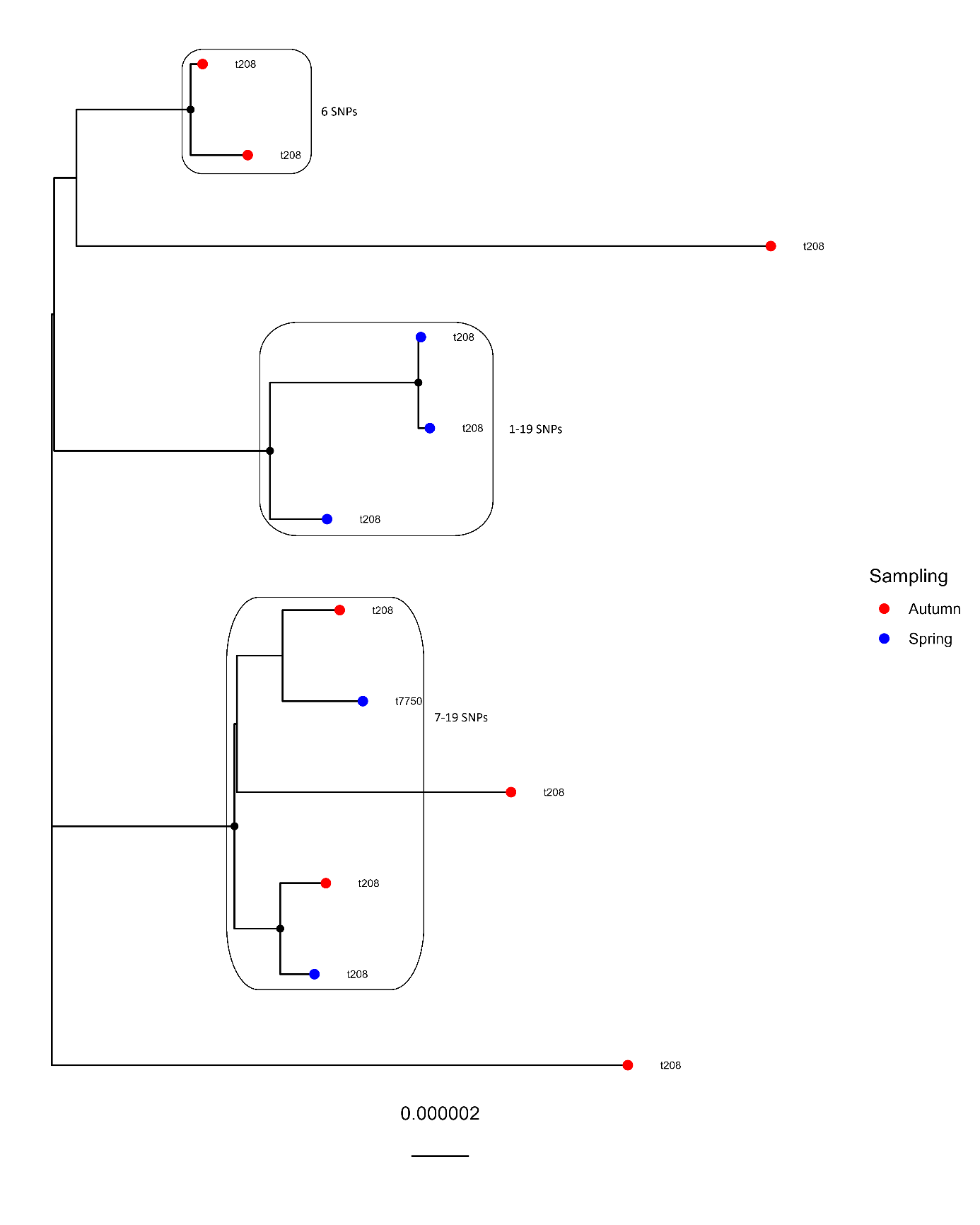


**Supplementary Figure 1**: Core genome SNP tree of 12 *Staphylococcus aureus* multilocus sequence type 49 isolates from ten different ewes at Farm A. Tip-points are coloured according to sampling occasion. Tip-labels indicate *spa*-type of the isolate. SNP ranges are indicated for closely related isolates.


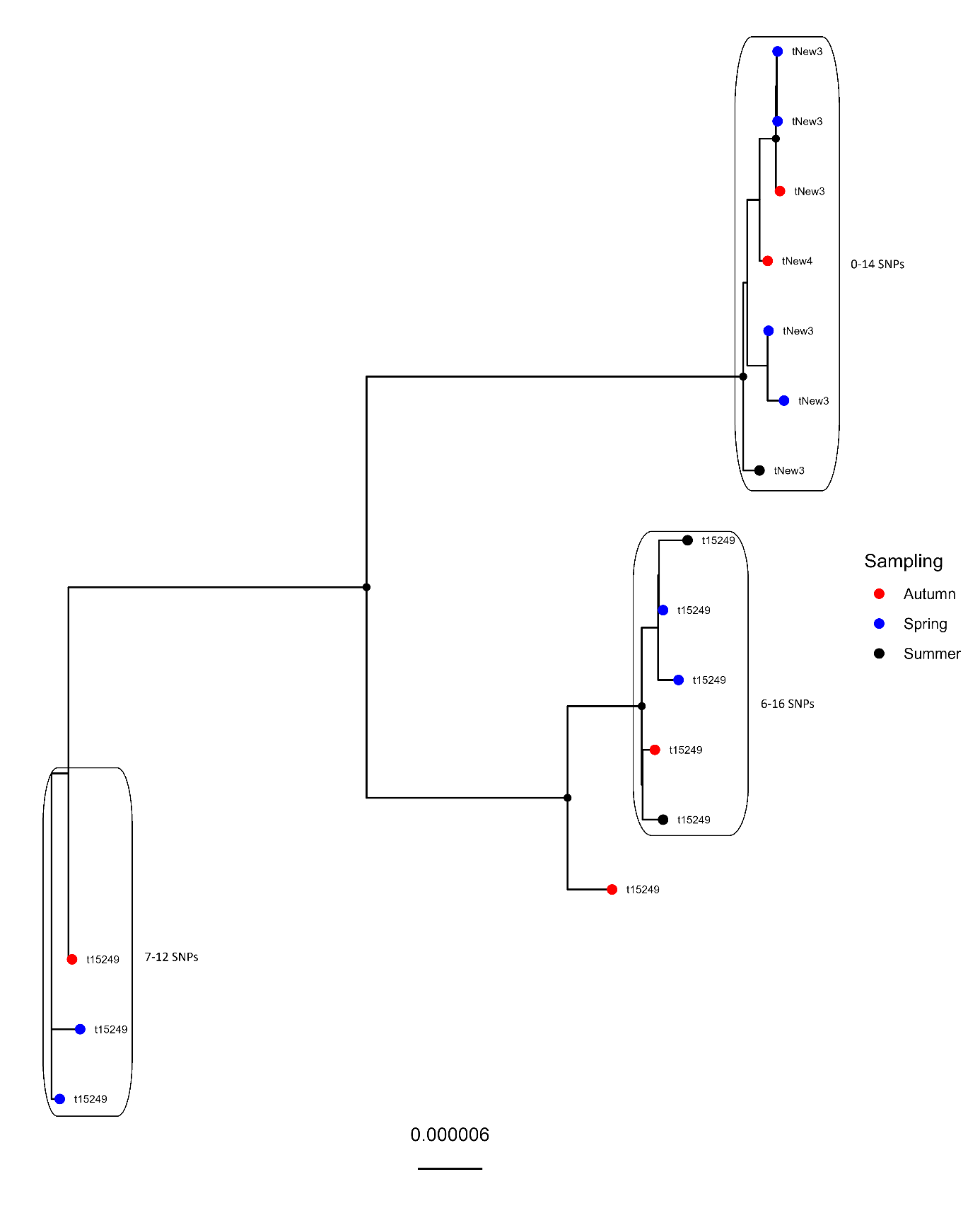


**Supplementary Figure 2:** Core genome SNP tree of 16 *Staphylococcus aureus* multilocus sequence type 133 isolates from 14 different ewes at Farm A. Tip-points are coloured according to sampling occasion. Samples collected during summer were from ewes with clinical mastitis. Tip-labels indicate *spa*-type of the isolate. SNP ranges are indicated for closely related isolates.


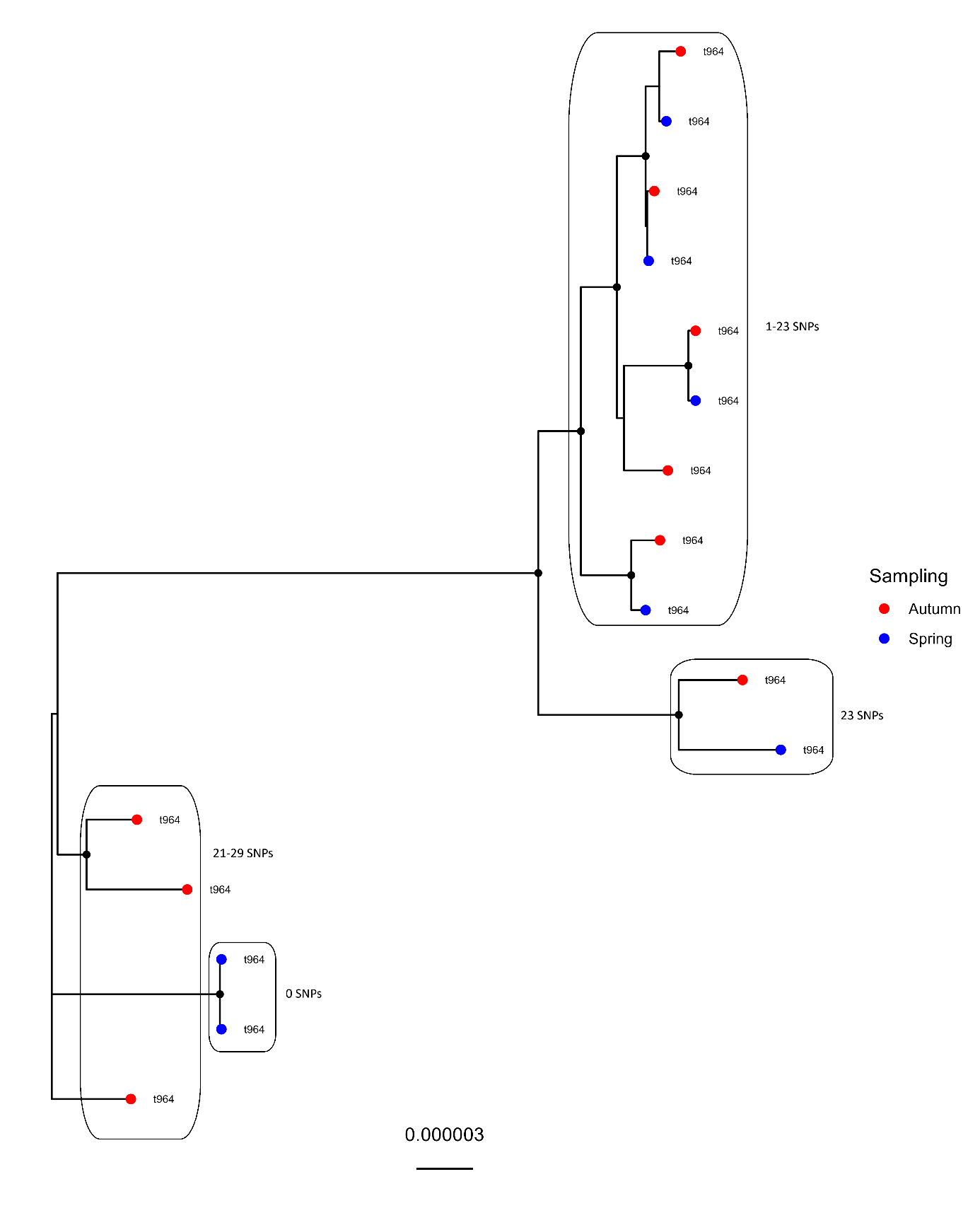


**Supplementary Figure 3:** Core genome SNP tree of 16 *Staphylococcus aureus* multilocus sequence type 30 isolates from eight different ewes at Farm B. Tip-points are coloured according to sampling occasion. Tip-labels indicate *spa*-type of the isolate. SNP ranges are indicated for closely related isolates.


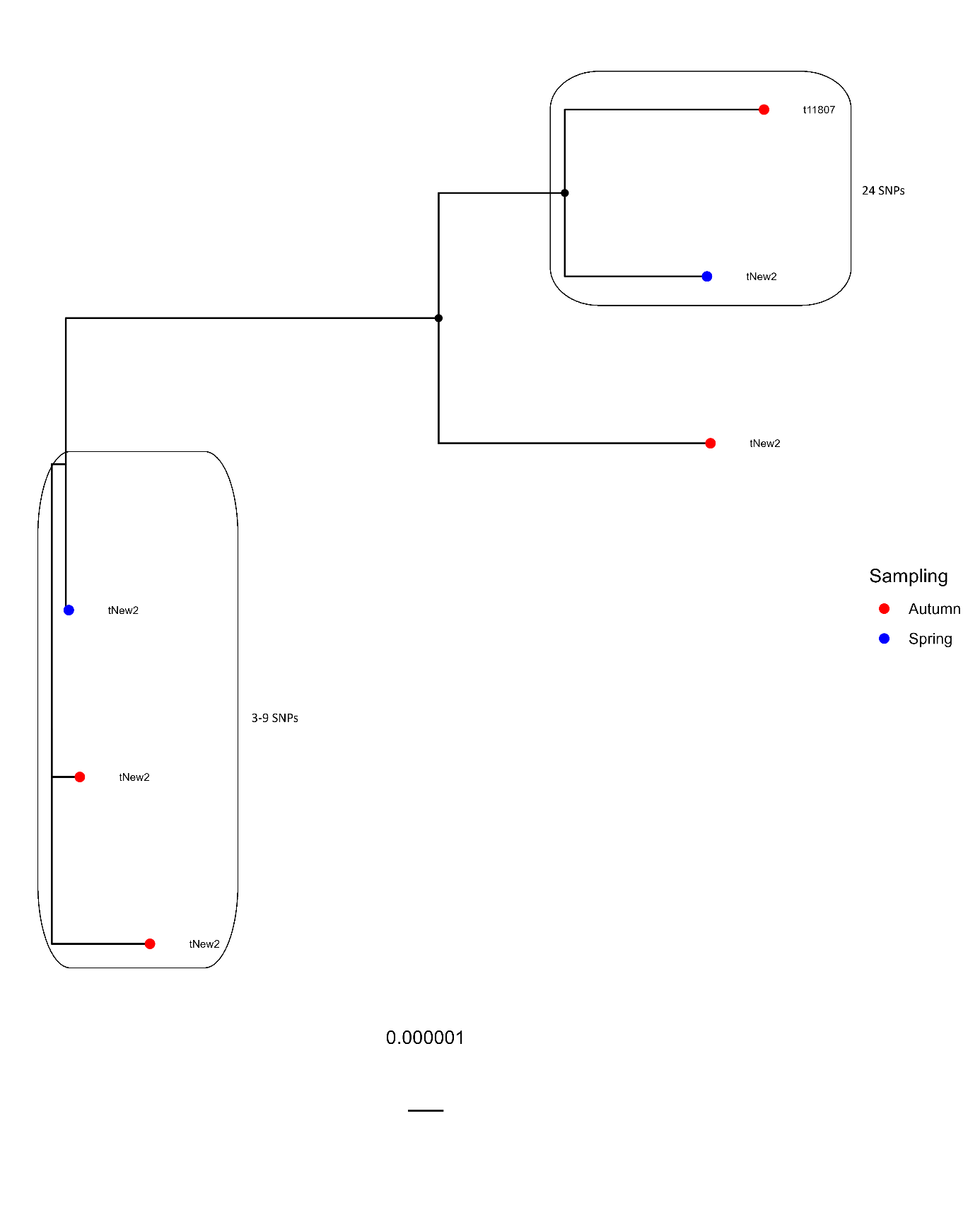


**Supplementary Figure 4:** Core genome SNP tree of six *Staphylococcus aureus* multilocus sequence type 49 isolates from four different ewes at Farm B. Tip-points are coloured according to sampling occasion. Tip-labels indicate *spa*-type of the isolate. SNP ranges are indicated for closely related isolates.


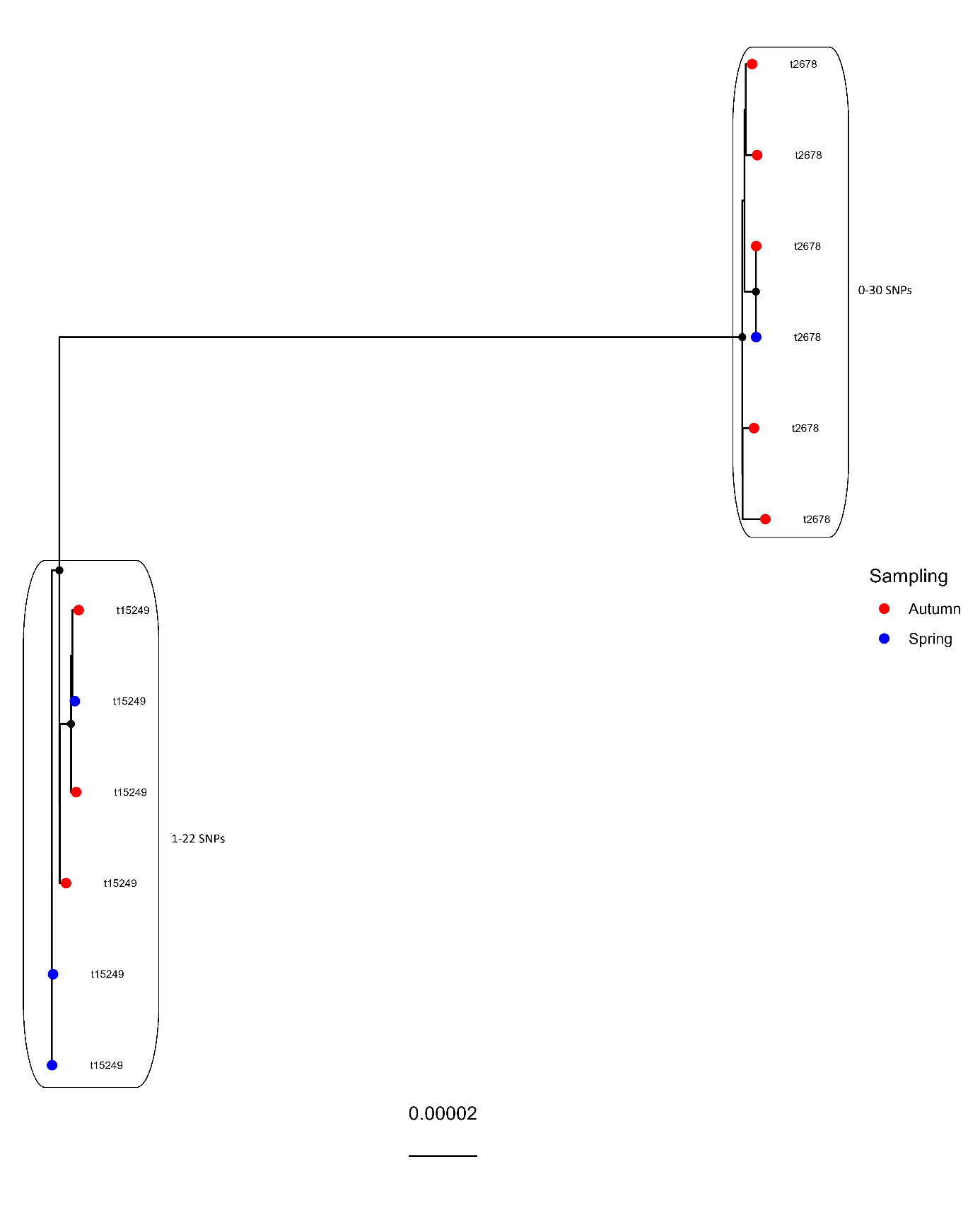


**Supplementary Figure 5:** Core genome SNP tree of 12 *Staphylococcus aureus* multilocus sequence type 133 isolates from ten different ewes at Farm B. Tip-points are coloured according to sampling occasion. Tip-labels indicate *spa*-type of the isolate. SNP ranges are indicated for closely related isolates.


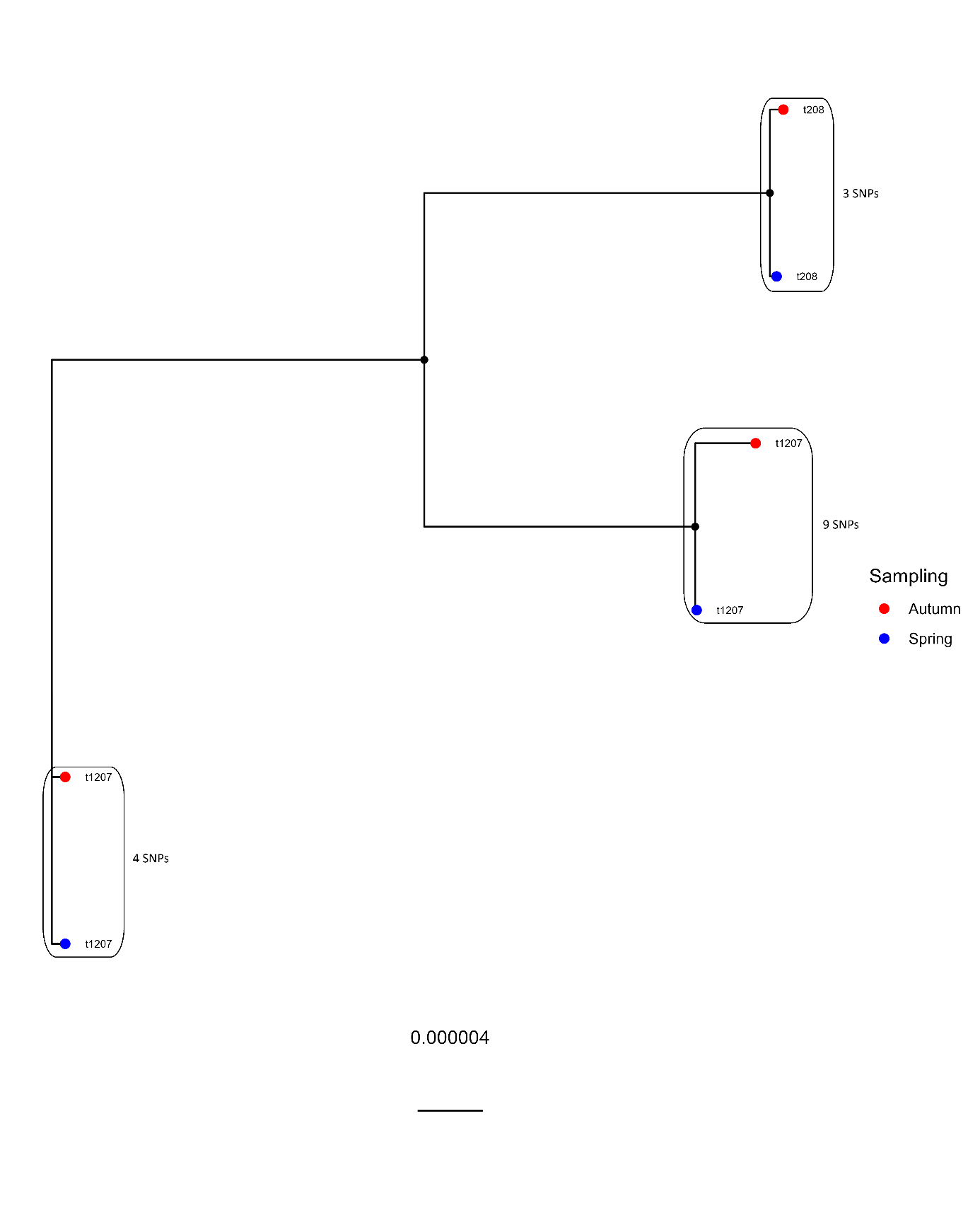


**Supplementary Figure 6:** Core genome SNP tree of six *Staphylococcus aureus* multilocus sequence type 49 isolates from three different ewes at Farm C. Tip-points are coloured according to sampling occasion. Tip-labels indicate *spa*-type of the isolate. SNP ranges are indicated for closely related isolates.


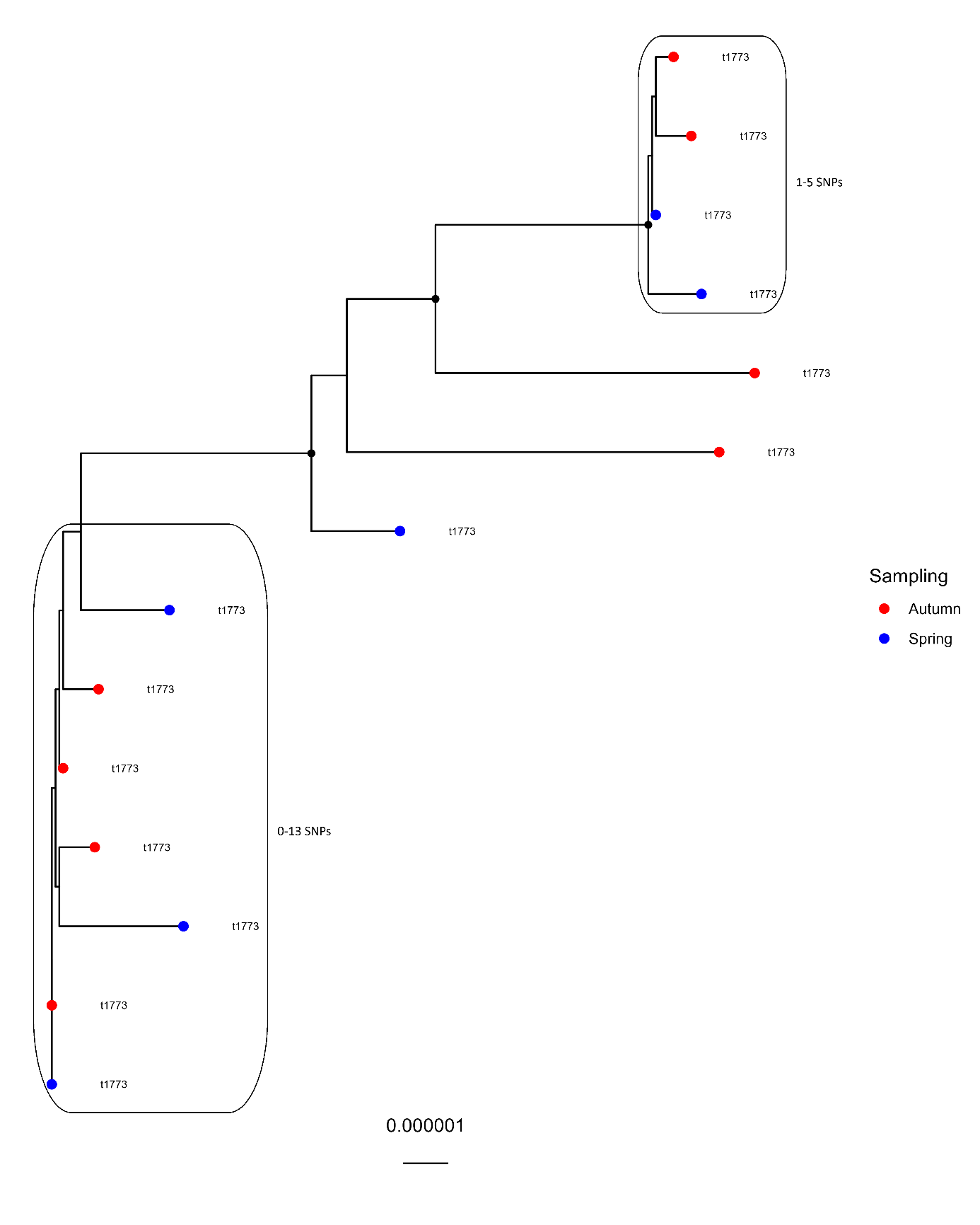


**Supplementary Figure 7:** Core genome SNP tree of 14 *Staphylococcus aureus* multilocus sequence type 130 isolates from 11 different ewes at Farm C. Tip-points are coloured according to sampling occasion. Tip-labels indicate *spa*-type of the isolate. SNP ranges are indicated for closely related isolates.


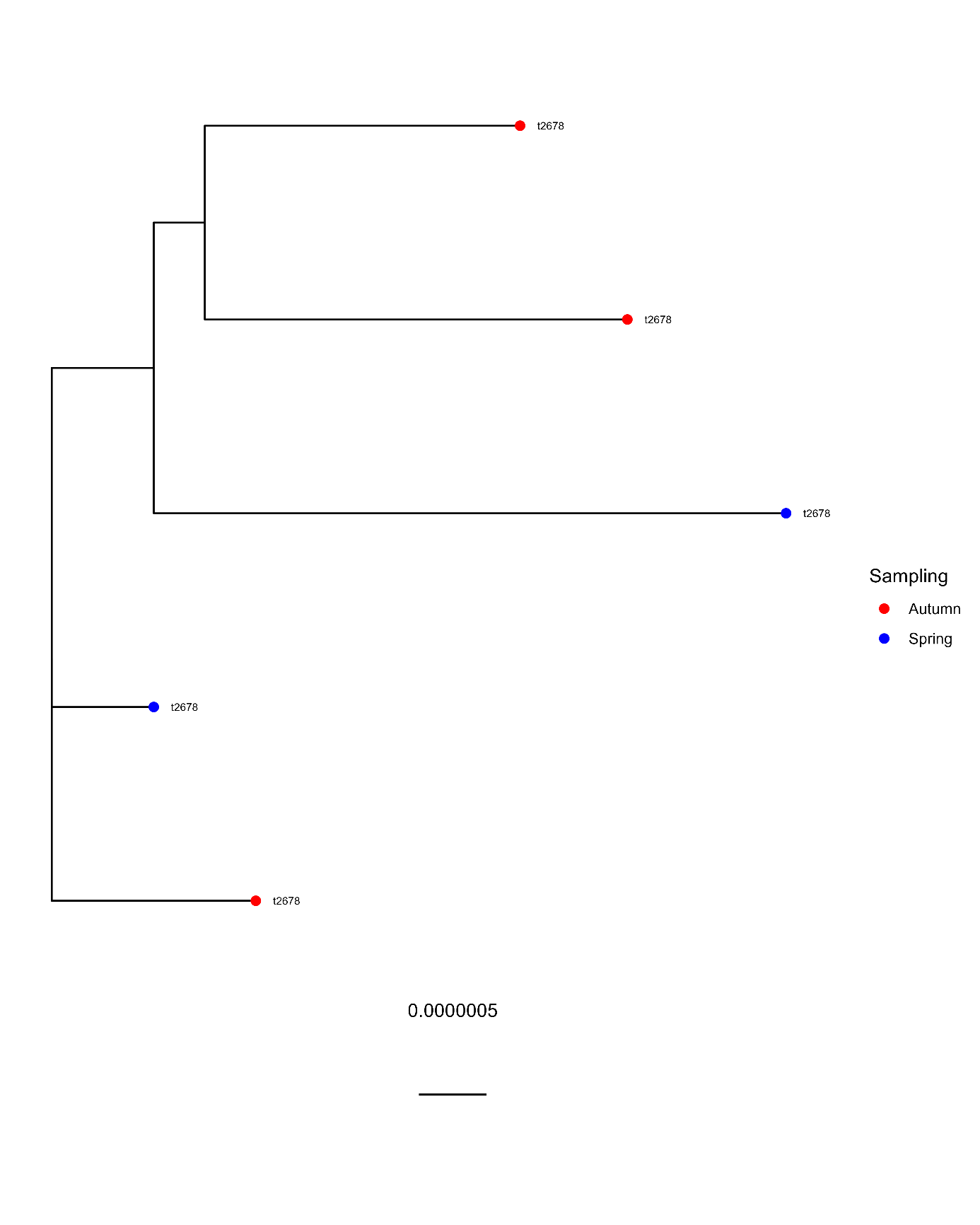


**Supplementary Figure 8:** Core genome SNP tree of five *Staphylococcus aureus* multilocus sequence type 133 isolates from four different ewes at Farm C. Tip-points are coloured according to sampling occasion. Tip-labels indicate *spa*-type of the isolate. SNP ranges are indicated for closely related isolates.


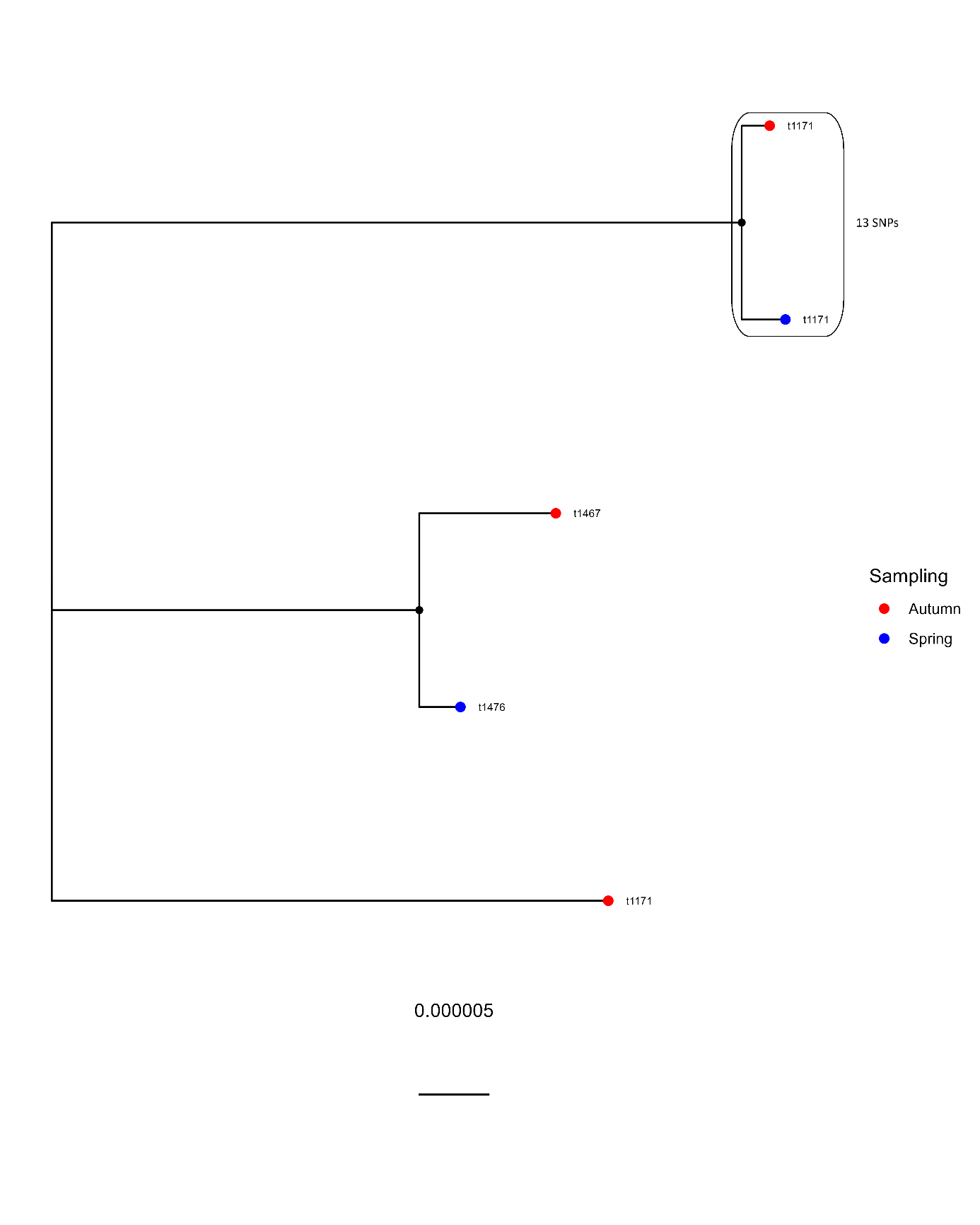


**Supplementary Figure 9:** Core genome SNP tree of five *Staphylococcus aureus* multilocus sequence type 8 isolates from three different ewes at Farm D. Tip-points are coloured according to sampling occasion. Tip-labels indicate *spa*-type of the isolate. SNP ranges are indicated for closely related isolates.

Supplementary Table QC
