# Supplementary material for: Genetic diversity and within-farm transmission of Staphylococcus aureus from ewes in Norway
Source: BMC Microbiol. 2026 Mar 20;26:412. doi: 10.1186/s12866-026-04959-z (PMC13126708; doi:10.1186/s12866-026-04959-z)
Supplement: Supplementary file 4 [file 12866_2026_4959_MOESM2_ESM.docx]

Supplementary Table 2: Minimum quality control (QC) requirements for raw sequencing reads of Staphylococcus aureus included in this study.

| QC parameter | Requirement |
| --- | --- |
| Mean per-base phred score | ≥ 20 |
| Coverage (C) calculated as read-length (L) divided by genome size (G) multiplied by number of reads (N): C=N*(L/G) | >30, genome size for S. aureus of 3 Mb used in calculation |
| Contamination | <5% of reads classified as another species |
| Average read length | Identical to expected read length from the sequencing platform used. |
